# Dual role for microbial short-chain fatty acids in modifying SIV disease trajectory following anti-α4β7 antibody administration

**DOI:** 10.1080/07853890.2024.2315224

**Published:** 2024-02-14

**Authors:** Samuel D. Johnson, Nageswara Pilli, Jianshi Yu, Lindsey A. Knight, Maureen A. Kane, Siddappa N. Byrareddy

**Affiliations:** a Department of Pathology and Microbiology, University of NE Medical Center, Omaha, NE, USA; b Department of Pharmacology and Experimental Neuroscience, University of Nebraska Medical Center, Omaha, NE, USA; c Department of Pharmaceutical Sciences, University of MD School of Pharmacy, Baltimore, MD, USA; d Department of Genetics, Cell Biology and Anatomy, University of Nebraska Medical Center, Omaha, NE, USA; e Department of Biochemistry and Molecular Biology, University of Nebraska Medical Center, Omaha, NE, USA

**Keywords:** Anti-α4β7 antibodies, SIV, short-chain fatty acid (SCFA), 16S rRNA, retinoic acid, gut

## Abstract

**Background:**

Human Immunodeficiency Virus (HIV)/Simian Immunodeficiency Virus (SIV) infection is associated with significant gut damage, similar to that observed in patients with inflammatory bowel disease (IBD). This pathology includes loss of epithelial integrity, microbial translocation, dysbiosis, and resultant chronic immune activation. Additionally, the levels of all-*trans*-retinoic acid (atRA) are dramatically attenuated. Data on the therapeutic use of anti-α4β7 antibodies has shown promise in patients with ulcerative colitis and Crohn’s disease. Recent evidence has suggested that the microbiome and short-chain fatty acid (SCFA) metabolites it generates may be critical for anti-α4β7 efficacy and maintaining intestinal homeostasis.

**Materials and Methods:**

To determine whether the microbiome contributes to gut homeostasis after anti-α4β7 antibody administered to SIV-infected rhesus macaques, faecal SCFA concentrations were determined, 16S rRNA sequencing was performed, plasma viral loads were determined, plasma retinoids were measured longitudinally, and gut retinoid synthesis/response gene expression was quantified.

**Results:**

Our results suggest that anti-α4β7 antibody facilitates the return of retinoid metabolism to baseline levels after SIV infection. Furthermore, faecal SCFAs were shown to be associated with retinoid synthesis gene expression and rebound viral loads after therapy interruption.

**Conclusions:**

Taken together, these data demonstrate the therapeutic advantages of anti-α4β7 antibody administration during HIV/SIV infection and that the efficacy of anti-α4β7 antibody may depend on microbiome composition and SCFA generation.

## Introduction

HIV infection is associated with significant disruptions of the gut mucosa, including damage to the gut epithelium, an overall loss of innate lymphoid cells (ILCs) with shifts from ILC3 to ILC1 phenotypes, and persistent CD4+ Th17 cell dysregulation [1–3]. This barrier breakdown facilitates increased microbial translocation, leading to chronic immune activation and, eventually, immune exhaustion, contributing to loss of virologic control and increased viral reservoir formation [2,4–6]. Further, this immune dysregulation contributes to a significant increase in disease burden, such as but not limited to HIV-associated neurocognitive disorders (HAND) [7]. Despite advances in combination antiretroviral therapies (cART), gut damage persists even following viral suppression [1]. In addition to loss of barrier function, significant microbial dysbiosis occurs during acute HIV infection and is never ameliorated despite the success of cART [8,9]. These disruptions reflect those that also occur during IBDs and include loss of *Bacteroides* and several butyrate-producing Firmicutes (notably *Roseburia* and *Faecalibacterium*) and an increase in pathogenic Proteobacteria taxa [8,10–15]. Together, these changes exacerbate gut damage and immune activation, further contributing to altered HIV pathogenesis, as in IBDs [10,11,16].

Similar to people living with HIV (PLWH), Simian Immunodeficiency Virus (SIV)-infected Asian macaques (genus *Macaca*) progress to immunodeficiency if untreated with cART [17,18]. However, several Old World monkey species native to Africa, such as sooty mangabeys (SM; *Cercocebus atys*) and African green monkeys (AGM; *Chlorocebus* spp.), are resistant to disease progression despite high viremia [19]. Interestingly, both disease-susceptible rhesus macaques (RM; *Macaca mulatta*) and pigtail macaques (PM; *Macaca nemestrina*) have significantly lower plasma all-*trans*-retinoic acid (atRA) concentrations than disease-resistant SMs [20]. In contrast, the relative expression of α4β7 integrin on diverse peripheral lymphocyte subsets from SMs was significantly lower than that on the same subsets from RMs or PMs [20]. Importantly, AGMs experience significant disruption of their gut during acute SIV infection but can rapidly initiate a pro-wound-healing immune response absent during both HIV and progressive SIV infections in RMs [21]. We hypothesized that there is a potentially essential role for α4β7 integrin expression in HIV/SIV disease progression.

atRA plays a pleiotropic role in the gut mucosa. Produced by both the intestinal epithelium and CD103+ dendritic cells (DCs), its generation and release signals for a significant immunomodulatory response, including the differentiation of pro-tolerogenic T-reg cells, IgA class-switching, and regulation of lymphocyte effector function [22]. Additionally, atRA induces the expression of both CCR9 and the aforementioned α4β7 integrin on diverse immune cell subsets [23]. One proposed mechanism for vedolizumab, a humanized anti-α4β7 mAb, is that blocking α4β7 integrin expressed on pro-inflammatory lymphocytes inhibits their trafficking to MAdCAM-1 expressed on high-endothelial venules of the gut, thereby preventing damage and allowing pro-healing responses during IBDs including Crohn’s disease (CD) and ulcerative colitis (UC) [24]. We recently proposed that the mechanism by which anti-α4β7 induces its therapeutic effect may be due to its ability to facilitate increased lamina propria macrophage maturity, which is typically lost during SIV pathogenesis. This relative increase in immature gut macrophages compared to mature gut macrophages that occurs during HIV/SIV infection may contribute to the chronic pro-inflammatory immune response [25,26].

To shed light on some of the above mechanisms, we quantified plasma and tissue retinoid and faecal SCFAs to elucidate the contribution of these factors. We found that cART facilitated the return of faecal SCFA levels following loss during acute infection. These levels were closely associated with bacteria taxa *Prevotella*, *Faecalibacterium*, and *Roseburia.* Further, subsequent SCFA concentrations predicted subsequent viral rebounds after analytic cART interruption. Our data also supports previous findings that anti-α4β7 facilitates the recovery of atRA synthesis. Faecal SCFA concentrations are closely associated with retinoic acid synthesis and response gene expression. Together, these data support two independent roles for microbial SCFAs in modulating HIV/SIV pathogenesis and the efficacy of anti-α4β7 antibody in facilitating a return to gut homeostasis.

## Materials and Methods

### Animal models and ethics statement

Nine female outbred Indian-derived RMs (aged 5.1 to 10.0 years old) were acquired from the Yerkes (since renamed Emory) National Primate Center of Emory University (Atlanta, GA, USA) and the New Iberia Research Center of the University of Louisiana (Lafayette, LA, USA) and transferred to the Department of Comparative Medicine at the University of Nebraska Medical Center (UNMC) (Omaha, NE, USA). RMs were maintained in accordance with the Animal Welfare Act and the Guide for the Care and Use of Laboratory Animals. Socialization was provided by pair housing and visual access to other RMs, and the climate was maintained at 72 °F with a 12-h light/dark cycle. RMs were provided a Purina monkey diet twice daily supplemented with fresh fruit and had continuous access to water. Anaesthesia was performed for all procedures with 10 mg/kg ketamine or 4 mg/kg telazol, and 0.2 mg/kg meloxicam was administered when deemed appropriate. Endpoint euthanasia was performed according to the American Veterinary Medical Association guidelines using high-dose ketamine-xylazine and exsanguination followed by cardiac perfusion. This study was planned, conducted, and reported here following the Animal Research: Reporting *In Vivo* Experiments (ARRIVE) guidelines. Ethical approval was obtained from the UNMC Institutional Animal Care and Use Committee (IACUC) and Institutional Biosafety Committee (IBC) for “Gut Trafficking Cells in SIV Infection” (Protocol #15-102-12) prior to initiation of the study as reported previously [25].

### Experimental design

**Figure 1.**
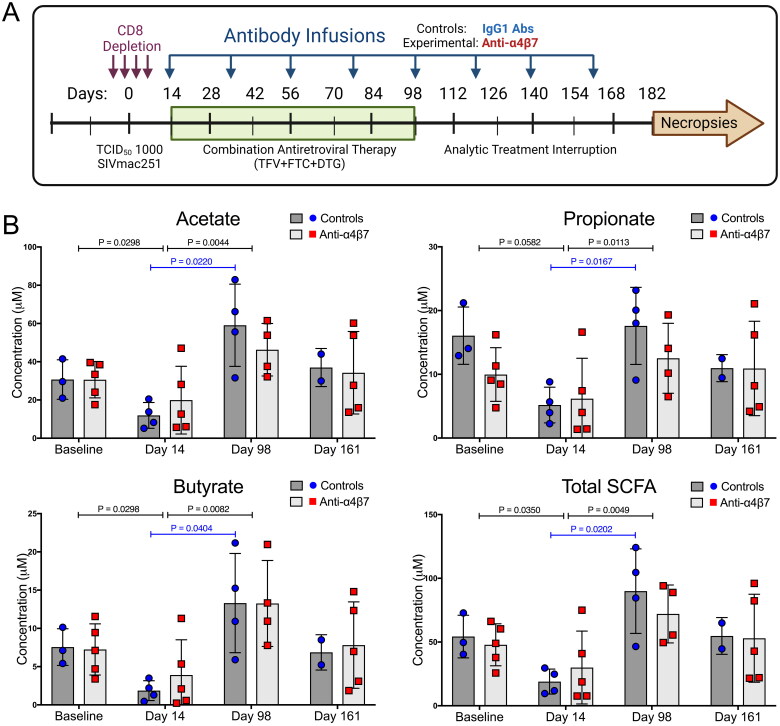
Antiretroviral therapy facilitates the recovery of faecal short-chain fatty acids following depletion during acute SIV infection. (A) Study schema. (B) Longitudinal concentrations of faecal SCFAs acetate, propionate, butyrate, and all three combined, as determined by liquid chromatography-tandem mass spectrometry. Statistical analyses were performed using unpaired T-tests when comparing between groups and paired T-tests when comparing within groups across time points (IgG1 Ab: *n* = 4; anti-α4β7 Ab: *n* = 5).

Baseline samples were acquired before study initiation at weeks −3 to −1. *In vivo* CD8+ cells were experimentally depleted by administration of an anti-CD8α IgG1 (MT807R1) (NIH Nonhuman Primate Reagent Resource (NHPRR)) subcutaneously (10 mg/kg on day −4) and intravenously (5 mg/kg on days −1, 3, and 6). The monkeys were intravenously infected with 1000 TCID_50_ of SIVmac251 (courtesy, Simian Vaccine Evaluation Unit, NIAID, NIH) on day 0. Therapy with cART (20 mg/kg TFV + 40 mg/kg FTC + 2.5 mg/kg DTG) was initiated on day 12 and continued daily until week 14. On day 15, RMs were randomly divided into two groups with *n* = 5 that were administered 50 mg/kg anti-α_4_β_7_ IgG1 mAb (A4B7, Lot: LH17-14-NIH) (NHPRR) and *n* = 4 a control IgG1 mAb (DSPR1, Lot: LH15-35) (NHPRR). Eight mAb infusions were administered regularly until week 23 (every three weeks). The protocol is shown in Figure 1(a) and was reported previously [25].

Sample collections included blood, gut tissue, and faecal samples. Blood collection was from the saphenous vein using K2-EDTA vacutainer tubes (Becton, Dickinson, San Diego, CA, USA) at baseline and on days 6, 10, 14, 28, 42, 77, 98, 105, 112, 119, 126, 140, and 161. Centrifugation was used to separate plasma from whole blood at 1200 rpm for 20 min, and the plasma was stored at −80 °C for further analysis. Faecal samples were collected at baseline and days 14, 98, and 161 using faecal loops and stored at −80 °C until analysis. At necropsy, duodenal and ascending colonic tissues were collected and snap-frozen. Aliquots of plasma, gut tissue, and faecal samples were sent to the University of Maryland (Baltimore, MD, USA) on dry ice to determine retinoid and SCFA levels.

### Plasma and tissue retinoid determination

Aliquots of plasma separated at baseline, days 14, 98, and 161 frozen and stored at −80 °C were then sent to the University of Maryland on dry ice. Snap-frozen gut tissue was also used to determine retinoid concentration. As previously described, retinoids were extracted under yellow light [27–29]. For plasma, 200 µL was extracted unless the volume was limited, and then either 80 µL or 150 µL was extracted. For the gut tissue, both the duodenum and ascending colon were analysed. For the duodenum, 26.6 − 177.2 mg (average 102.5 ± 40.2 mg) of tissue was homogenized and extracted. For ascending colon, 45.0 − 132.6 mg (average 77.6 ± 29.1 mg) of tissue was homogenized and extracted. Tissue was homogenized in 1.0 mL saline (0.9% NaCl) with ground glass homogenizers (Kontes, size 22), as previously described [29,30]. Concentrations of retinyl ester (RE) and retinol (ROL) were determined using HPLC-UV with an ACQUITY H-Class UPLC equipped with a PDA detector (Waters, Milford, MA). Concentrations of atRA were determined by liquid chromatography-multistage-tandem mass spectrometry using atmospheric pressure chemical ionization in positive-ion mode with a 6500+ QTRAP hybrid tandem quadrupole mass spectrometer (AB Sciex, Foster City, CA) [31]. Plasma retinoids are expressed as moles per milliliter, and tissue retinoids are defined as moles per gram of tissue.

### Duodenal and colonic gene expression

Gut tissue RNA was isolated from frozen tissues using an AllPrep DNA/RNA Micro Kit (Qiagen GmbH, Hilden, Germany; Product #80284). cDNA was synthesized using SuperScript™ IV Reverse Transcriptase (Thermo Fisher Scientific Baltics UAB, Vilnius, Lithuania; Product # 18090050), as recommended by the manufacturer. PowerUp™ SYBR™ Green Master Mix (Thermo Fisher Scientific Baltics UAB, Vilnius, Lithuania, Product #A25742) was used for real-time PCR according to the manufacturer’s instructions. Primers were generated using the NCBI tool Primer-BLAST using mRNA transcript sequences from NCBI Gene for RMs (Organism:9544). Primers were designed to span exon–exon junctions, have a melting temperature of 60 °C to 63 °C, and have an amplicon length of 70 to 200 base pairs. Primer sequences are shown in Supplemental Table 1. Gene expression was normalized to that of GAPDH and expressed relative to the geometric means of all samples.

### Faecal short-chain fatty acid concentrations

The quantification of endogenous short-chain fatty acids (SCFA) in faeces was carried out using liquid chromatography-tandem mass spectrometry (LC-MS/MS), as previously described [32]. Butyric acid (BA), acetic acid (AA), and propionic acid (PA) were quantified using stable isotope-labelled internal standards (butyric acid-d7, acetic acid-d3, and propionic acid-d5). Briefly, 100 mg of faecal sample was homogenized in 50% aqueous acetonitrile to obtain a 20 mg/mL homogenate. Homogenates were vortexed mixed for 5 min and then centrifuged at 4000× *g* at 10 °C for 10 min to extract SCFAs. 10 µL of the supernatant was added to 10 µL of N-(3-dimethylaminopropyl)-N’-ethyl-carbodiimide hydrochloride (EDC; 120 mM in 6%pyridine in acetonitrile), 10 µL of 3-NPH (200 mM in acetonitrile) and 10 µL of internal standard solution (100 µM of butyric acid d7, 2 mM of acetic acid d3 and 100 µM of propionic acid d5). This mixture was incubated for 15 min at 40 °C, then diluted with acetonitrile to a volume of 1 mL. The samples were chromatographed on a Phenomenex Kinetex C18 (2.1 × 100 mm, 2.6 µm) column using 0.01% formic acid in water (A) and 0.01% formic acid in acetonitrile (B) as the mobile phase with a 4 min gradient program followed by detection according to unique *m/z* transitions on an Altis tandem quadrupole mass spectrometer using electrospray ionization operated in negative ion mode (Thermo, San Jose, CA).

### Plasma viral loads

Plasma samples were isolated from whole blood samples following centrifugation at 1200 rpm for 20 min. RNA was isolated by spin column chromatography using a QIAamp Viral RNA Mini Kit (Qiagen, Germantown, MD, USA; Product #52906). As previously described, viral loads (VLs) were determined by real-time PCR using TaqMan® RNA-to-Ct™ 1-Step Kit (Thermo Fisher Scientific Baltics UAB, Vilnius, Lithuania, Product #4392938), and standard curves were evaluated in parallel [25,33,34]. Real-time PCR was performed for 40 cycles with a 1-min annealing/extension step at 60 °C. The primers used were fwd: CATCAAGCAGCCATGCAAT and rev: ATCTGGCCTGATGCAATAG.

### 16S rRNA sequencing

16S rRNA analysis was performed, and the relative abundances of phyla and families were determined, as reported previously [25]. Briefly, faecal samples were thawed and DNA was isolated by spin column chromatography as previously described [25,35]. DNA was shipped to LC Sciences, LLC (Houston, TX, USA) on dry ice for 16S rRNA gene sequencing. The V3 and V4 variable regions were amplified for library generation, and an Illumina cBot system was used for clustering. Illumina Mi Seq was used to perform sequencing, and demultiplexed sequences were annotated using RDP, Greengenes, and NCBI 16SMicrobial customized databases. Data output statistics, including operational taxonomic unit clustering, taxonomic classification, and relative abundance, were determined by LC Sciences. All data presented herein are limited to genus ranking, which had not been previously reported, and were utilized for comparison with faecal SCFA concentrations. The generated FASTQ files were uploaded to the NCBI Sequence Read Archive and are available with BioProject (NCBI BioProject, RRID:SCR_004801) accession number PRJNA870961 [25].

### Statistical analysis

**Figure 2.**
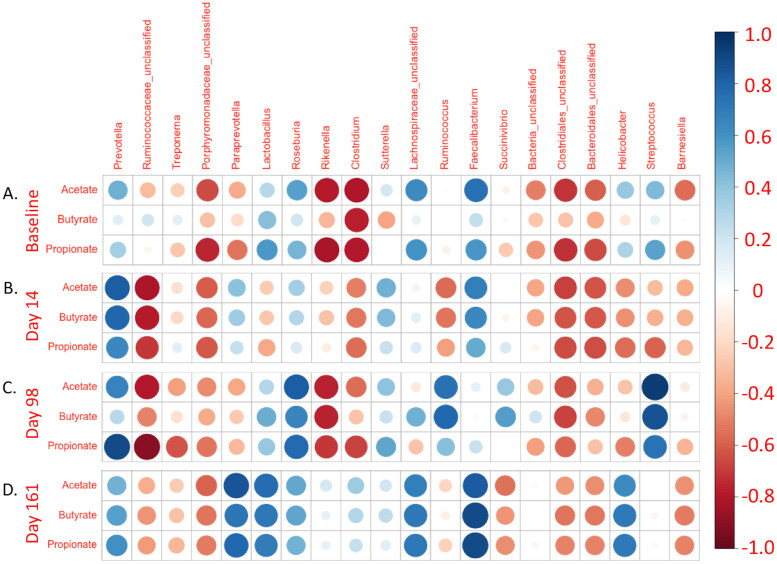
Faecal SCFA concentrations are associated with diverse faecal microbiome taxa. Modified correlogram comparing (A) baseline SCFA concentrations with baseline relative abundance of the twenty most abundant faecal microbiome genera. Days (B) 14 (acute infection), (C) 98 (last day of cART), and (D) 161 (nine weeks ATI) faecal SCFA concentrations were also compared with respective timepoint genus relative abundances from the faecal microbiome. Pearson coefficients and P-values are available in Supplemental Table 2.

Unpaired T-tests were used to compare the experimental groups. Paired T-tests were used to compare the time points within groups. The averages were determined by calculating geometric means. Linear regression was performed to determine the associations between independent and dependent variables. Statistical significance was defined as *p* < 0.05. All statistical analysis was performed using GraphPad Prism 7 (GraphPad Prism, RRID:SCR_002798) for Mac OS X. Further analysis was performed with RStudios R version 3.5.0 [36]. Figures 2 and 4 are modified correlograms (cropped for size and redundancy) generated using the R package ‘corrplot’ Version 0.92 [37].

## Results

### Combination antiretroviral therapy is associated with reversal of decreased levels of faecal short-chain fatty acid following loss during acute infection

To determine whether SCFA production plays a role in anti-α4β7 efficacy, as suggested in our previous report [25], we quantified the concentrations of the most abundant SCFAs in the samples obtained at baseline and on days 14, 98, and 161. Consistent with current models, SCFA concentrations trended lower during acute infection (Figure 1(b)). However, following cART and several antibody administrations (day 98), both groups tended to show higher levels of SCFAs, with only the control group showing increased values that were statistically significant from acute infection (Figure 1(b)). This was true for all SCFAs analysed and for total SCFA concentrations. Because both groups rebounded from low levels of SCFA during acute infection, there were no significant differences between the groups. However, when both groups were combined, acute infection was associated with a significant decrease in the levels of acetate, butyrate, and total SCFAs and subsequent restoration (a significant increase from days 14 to 98) of these lost SCFAs (as well as a significant increase in propionate) following cART administration.

### Faecal microbiome composition is associated with changes in the levels of faecal short-chain fatty acids

To better understand the interrelationship between faecal SCFAs and microbiome composition, 16S rRNA gene sequencing was performed, and genus identity was assigned (Figure S1). Next, linear regression was performed, in which the 20 most abundant genera were compared with each SCFA reported above (Figure 2, Table S2). Analysis was limited to these 20 because individual genera were not detected in many samples after them, thus limiting the appropriateness of linear regression (where relative abundance would be 0%). 34 pairings were found with a significance cutoff of *p* = 0.05, a finding much higher than that expected by chance (Figure 2, Table S2). Further, many of these genera comprised a substantial percentage of individual reads, leading to high relative abundances. They are, therefore, likely able to contribute significantly to the metabolism of dietary fiber to SCFAs. Eight genera had zero associations with faecal SCFAs at any point in time. Two genera had one association, three had two associations, and four had three associations. Three genera *Rikenella*, *Ruminococcaceae_unclassified*, and *Faecalibacterium* had more than three associations, suggesting essential interrelationships between these genera and SCFA production. *Rikenella* was negatively associated with acetate and butyrate concentrations in the samples obtained at baseline and day 98. Similarly, *Ruminococcaceae_unclassified* was negatively associated with acetate, propionate, and butyrate in samples on day 14 and negatively associated with acetate and propionate in samples on day 98. In contrast, *Faecalibacterium*, a butyrate-producing taxon frequently associated with improved clinical outcomes in several diseases, was positively associated with acetate in samples on day 14 and with acetate, propionate, and butyrate in samples on day 161. When a Bonferroni correction was applied to each analysis (*p* < 0.0025), two associations remained. *Streptococcus* was positively associated with acetate at day 98, and *Ruminococcaceae_unclassified* was negatively associated with propionate also at day 98.

Two additional taxa were previously reported from this study as a proxy for HIV/SIV-associated dysbiosis [29]. These include *Roseburia*, which was unexpectedly positively associated with acetate and propionate (but not butyrate) in samples obtained on day 98, and *Prevotella*, which was positively associated with acetate and butyrate in samples obtained on day 14 and propionate on day 98. Overall, these findings suggest that the composition of the microbiome is more critical for SCFA production than any specific taxon. Furthermore, these findings provide evidence that faecal SCFAs are not necessarily associated with their presumed producers, as has been extensively discussed in the literature on HIV/SIV-associated dysbiosis, suggesting that butyrate-producing bacteria (BPB) may have alternative roles in modulating HIV and IBD pathogenesis [10,29,49].

#### Levels of faecal short-chain fatty acids are predictive of subsequent plasma viral loads

**Figure 3.**
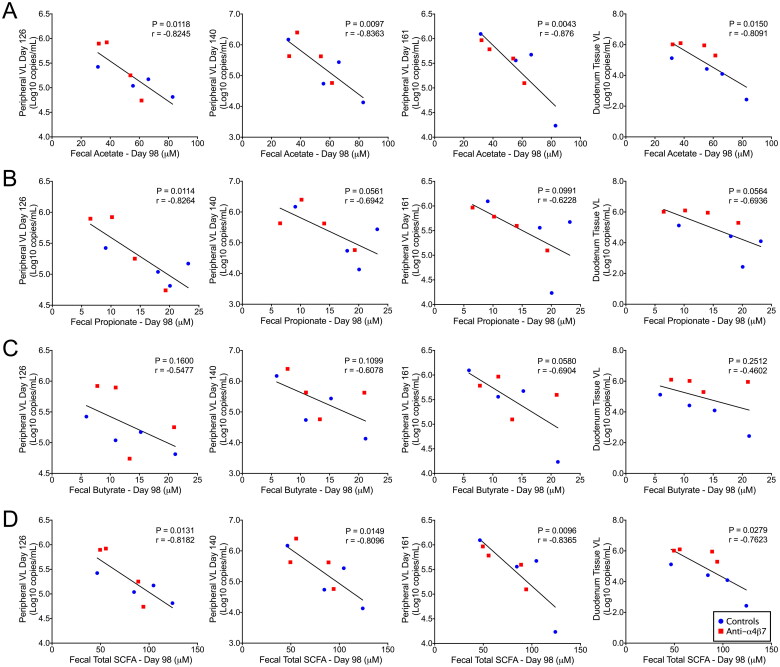
Faecal short-chain fatty acids at day 98 predicted rebound viral loads after analytic cART interruption. Linear regression was used to compare SCFAs (A) acetate, (B) propionate, (C) butyrate, and (D) total SCFAs with rebound viral loads at days 126, 140, and 161, as well as duodenal tissue viral loads determined at necropsy. Statistical correlation analysis was performed to determine Pearson’s coefficient.

In the present study, all RMs were experimentally depleted of CD8+ cells, which has previously been shown to increase viral loads and reduce viral control [38]. In line with this finding, all RMs in this study rebounded with positive VLs following cART interruption (Figure S2). These rebound VLs were subsequently compared with faecal SCFA concentrations, and important associations were observed. Consistent with our previously posited hypothesis, rebound VLs were negatively associated with SCFA concentrations on day 98 during mAb administration (Figure 3) [25]. Faecal acetate was negatively associated with plasma VLs in samples obtained on days 126, 140, and 161 (Figure 3(a)). Faecal acetate was also negatively associated with previously reported duodenal tissue VLs during necropsy. Similarly, propionate levels on day 98 faecal samples were negatively associated with rebound plasma VLs at day 126, with trends at days 140 and 161, and in duodenal tissue VLs (Figure 3(b)). Surprisingly, and contrary to our current understanding of HIV/SIV pathogenesis, faecal butyrate concentrations were not associated with VL rebounds (Figure 3(c)) [10,25,39]. Combined SCFAs had similar associations as acetate (Figure 3(d)), an unsurprising finding because acetate comprised the bulk of SCFAs measured in this study. Together, these data suggest that acetate, and not butyrate, as previously indicated by us and others, is predictive of the magnitude of viral rebound during ATI.

### Anti-α4β7 mAb infusions facilitate retinoid metabolism recovery

**Figure 4.**
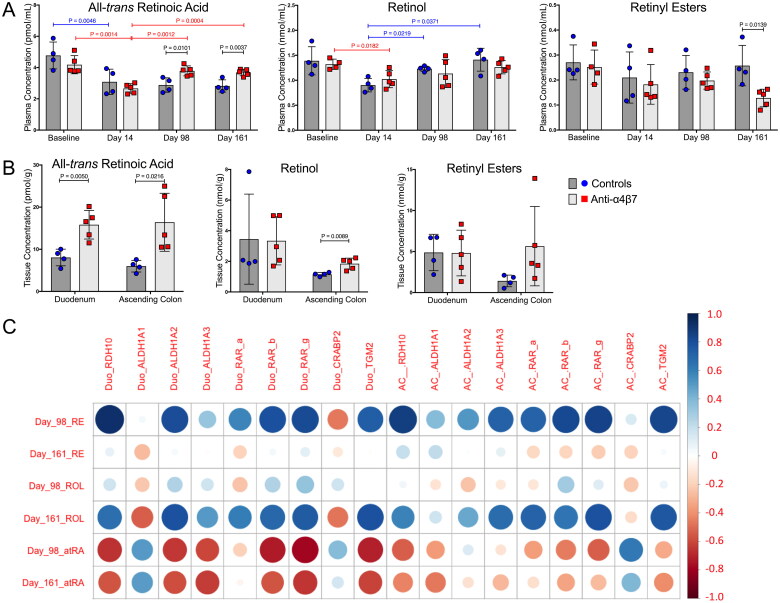
Plasma and gut all-*trans*-retinoic acid metabolism are rescued by anti-α4β7 administration. (A) Quantification of plasma retinoids atRA, ROL, and RE concentrations. (B) Gut tissue plasma retinoid concentrations in the duodenum and ascending colon at necropsy. Statistical analyses were performed using unpaired T-tests when comparing between groups and paired T-tests when comparing within groups across time points (IgG1 Ab: *n* = 4; anti-α4β7 Ab: *n* = 5). (C) Modified correlogram comparing plasma retinoids at days 98 and 161 with gut gene expression at necropsy. Pearson correlation coefficients and P-values were also determined and are available in Supplemental Table 3.

During SIV infection, atRA levels are rapidly depleted concurrently with gut damage [40]. We previously reported that anti-α4β7 treatment facilitated the recovery of myeloid cell phenotypes lost during SIV infection, raising the possibility of a contributory role for myeloid cells in retinoid metabolism recovery, similar to previous reports [25,40]. To test this hypothesis, biologically relevant retinoids were quantified in the RM plasma across several time points, including baseline (prior to CD8 depletion and infection), day 14 (acute infection), day 98 (therapy), and day 161 (63 days post-cART interruption) (Figure 4(a)). Consistent with results from previous studies, acute infection was associated with a significant decrease in plasma atRA levels in both control and anti-α4β7-treated groups [22,40]. Plasma atRA levels remained depressed in the control animals during subsequent sampling. However, unlike the control groups, anti-α4β7-administered RMs had increased levels of atRA on days 98 and 161 compared to day 14. This increase following therapy showed significant differences between the control and anti-α4β7-treated RMs at days 98 and 161. Next, the plasma retinoids upstream of atRA synthesis were quantified (Figure 4(a)). The plasma ROL decreased in both groups on day 14. This precursor of atRA, however, significantly increased in the control group but not in the anti-α4β7 group from low values in samples from day 14, suggesting that untreated macaques had atRA precursor levels that returned to baseline with no impact on the reduced atRA levels, indicating that precursors may have remained unutilized for atRA synthesis. No significant differences were observed between the time points for plasma RE levels. However, control RMs had significantly higher RE on day 161, indicating that control animals do not lack precursors of atRA.

Anti-α4β7 mAb therapy was developed to modulate the trafficking of immune cells to ameliorate damage from gut inflammation [24]. Because we found differences in gut myeloid cells in our previous study [25], we characterized retinoid concentrations in the duodenum and ascending colon at necropsy (Figure 4(b)). The atRA level was significantly higher in both the duodenum and ascending colon in anti-α4β7 antibody treated RMs than in controls. Unexpectedly, ROL was also markedly higher in the ascending colons of treated macaques, and similarly, there was a trend in RE as well. These findings suggest significant improvements in active metabolite (atRA) levels and maintenance and/or increased substrate levels for atRA synthesis in the guts of anti-α4β7antibody treated RMs during SIV infection.

Much of the above data implicate gut myeloid cells as mediators of increased plasma and gut atRA following anti-α4β7 antibody administration. To determine whether previously acquired data support this proposed mechanism, linear regression analysis was performed with reported markers of gut macrophage maturation, namely the co-localization of CD206 with CD163 in the lamina propria [25,26]. We previously reported a significant increase in the expression of gut macrophage maturation markers, as determined by flow cytometry- and microscopy-derived evidence [25]. Here, we found that the Mander’s coefficient (ratio of the co-localization of CD206 and CD163 compared with CD163 alone) in the duodenum was positively associated with plasma atRA at days 98 and 161, as well as duodenal tissue atRA at necropsy, adding further evidence that a return to small intestinal immune homeostasis typically lost during SIV/HIV infection may be driving both systemic and tissue increases in retinoid metabolism (Figure S3).

Next, to better understand the regulation of retinoid signaling in the gut, various retinoic acid synthesis (RDH10, ALDH1A1, ALDH1A2, and ALDH1A3) and response genes (RAR-a, RAR-b, RAR-c, CRABP2, and TGM2) that had previously been identified for their relationship to microbial response were quantified using real-time qPCR (Figure S4) [41]. No significant differences were observed in any of the genes examined. However, there were trends in the decreased expression of selected genes involved in atRA synthesis. These included genes for RDH10, ALDH1A2, and ALDH1A3 in both tissues during anti-α4β7 therapy compared with controls. These findings are consistent with our previous report suggesting that anti-α4β7 administration was associated with a decrease in CD103+ conventional dendritic cells, which are canonically known to release retinoic acid to modulate the mucosal immune response [25]. Additionally, there was a trend toward a reduction in the expression of all retinoic acid response genes, except for CRABP2, for which there was a trend of increased expression.

To further elucidate retinoid metabolism dynamics, gut gene expression in tissues obtained at necropsy was compared to that in tissue retinoids (Figure 4(c), Table S3). Interestingly, plasma RE at day 98, following multiple administrations of either anti-α4β7 or control IgG, was predictive of subsequent retinoid synthesis and retinoid response genes in both the small and large intestine. This included positive associations with atRA synthesis genes such as RDH10 and ALDH1A2 and atRA response genes RAR-b, RAR-g, and TGM2 in the duodenum and RDH10, ALDH1A3, RAR-a, RAR-b, RAR-g, and TGM2 in the ascending colon (Figure 4c, Table S3). Similarly, a positive association was found between plasma ROL on day 161 and ALDH1A2 (but not RDH10) and the response genes RAR-b, RAR-g, and TGM2 in the duodenum in samples obtained at necropsy. Likewise, a positive association was found between ROL at day 161 and the expression of ALDH1A3 and the response genes RAR-a, RAR-g, and TGM2 in samples from the ascending colon (Figure 4(c), Table S3).

### Short-chain fatty acids are associated with atRA synthesis and response genes

**Figure 5.**
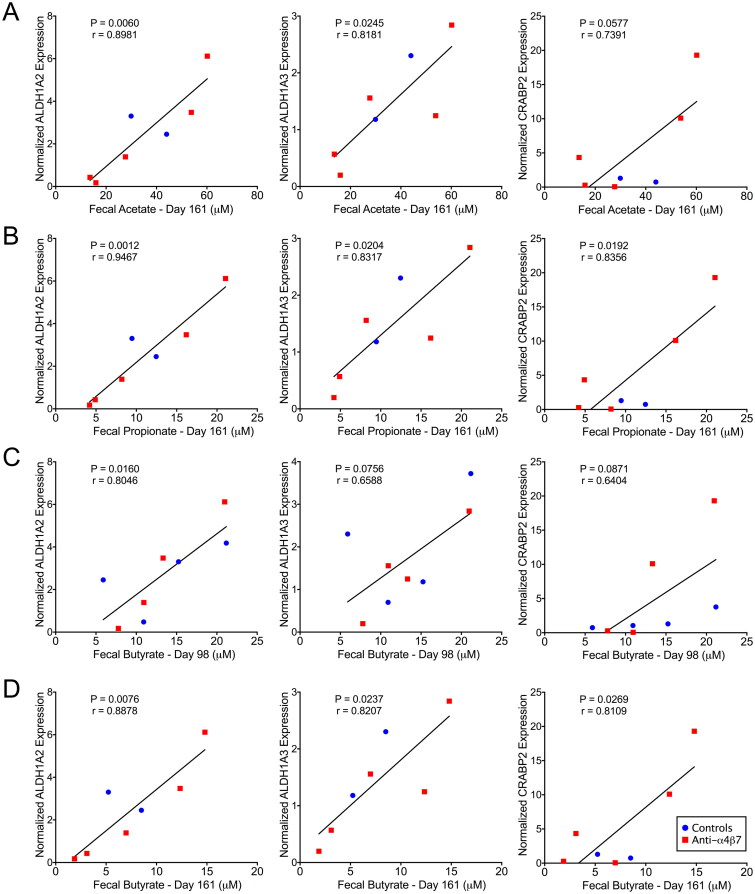
Faecal short-chain fatty acids are associated with retinoid metabolism genes. atRA synthesis and response genes in the ascending colon were compared with longitudinal faecal SCFAs with linear regression. Statistical correlation analysis was performed to determine Pearson’s coefficient, and significant associations were found between ALDH1A2, and ALDH1A3 were found with day 161 (A) acetate and (B) propionate; ALDH1A2 with day 98 (C) butyrate; and ALDH1A2, ALDH1A3, and CRABP2 with day 161 (D) butyrate.

**Figure 6.**
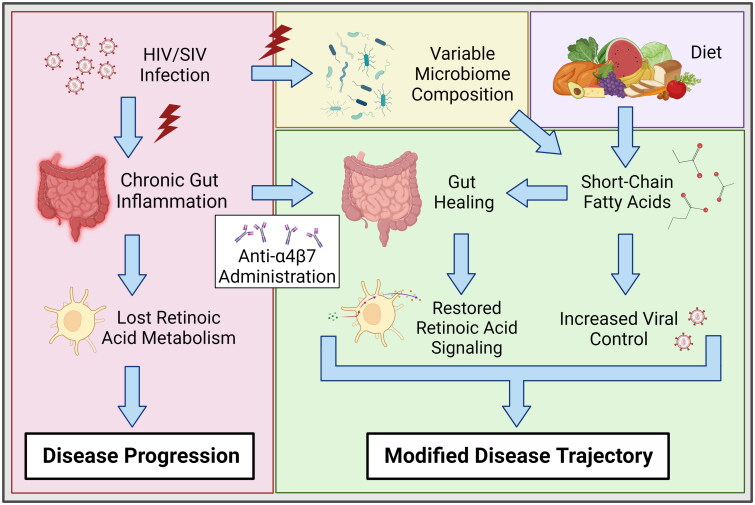
Role of SCFAs on modifying anti-α4β7 therapeutic efficacy. HIV/SIV infections are associated with significant gut damage and chronic inflammation, resulting in substantial attenuation of atRA metabolic homeostasis. Anti-α4β7 mAbs can facilitate gut healing and subsequently improve atRA concentrations. Importantly, faecal SCFA concentrations were associated with atRA synthesis and response genes. Additionally, SCFAs are negatively associated with viral loads, suggesting they facilitate improved viral control. Figure created with BioRender.com.

Previous data have indicated that SCFAs, particularly butyrate, are essential for maintaining the cell types required for synthesizing atRA and for maintaining pro-regulatory phenotypes in gut immune cells [42–46]. To better understand the role of SCFAs in atRA signalling in our model, we performed linear regression on the quantified genes and faecal SCFAs (Figure 5). ALDH1A2, ALDH1A3, and CRABP2 were frequently positively associated with faecal SCFA levels after therapy. Acetate was associated with ALDH1A2 and ALDH1A3, and propionate was associated with ALDH1A2, ALDH1A3, and CRABP2 in samples obtained on day 161. Neither of these SCFAs was significantly related to subsequent gene expression on day 98. In contrast, butyrate on day 98 was associated with ALDH1A2 expression. Trends were also observed for ALDH1A3 and CRABP2 expression. Butyrate was significantly associated with ALDH1A2, ALDH1A3, and CRABP2 on day 161. These data suggest that SCFAs, and perhaps butyrate specifically during therapy, are essential for subsequent retinoid metabolism. This finding builds on previous data showing that the microbiome and its metabolites are critical determinants of anti-α4β7 antibody efficacy (Figure 6) [25,47].

## Discussion

The bacterial constituents of the microbiome have been heavily implicated in HIV/SIV pathogenesis, contributing to acute and chronic immune activation following acquisition. Characteristic changes include the loss of butyrate-producing Firmicutes (recently renamed Bacillota following the elimination of phylum ranking by the International Committee on Systematics of Prokaryotes and subsequent adoption by NCBI) and *Bacteroides* within Bacteroidetes (renamed Bacteroidota) [8,13,14], which are accompanied by increases in Proteobacteria (renamed Pseudomonadota) and the Bacteroidete *Prevotella* [8,11,13–15]. Unfortunately, without intervention, HIV/SIV-associated dysbiosis persists even during cART [8,9]. The loss of butyrate-producing genera, such as *Blautia*, *Roseburia*, and *Faecalibacterium*, has suggested that the loss of faecal butyrate leads to the persistence of gut damage [9]. During homeostasis, butyrate contributes to gut barrier function, acts as an HDAC1 inhibitor, and is an agonist of GPR41, GPR43, and GPR109 [42,43]. These butyrate functions have been hypothesized to facilitate a return to immune homeostasis and reduce the viral reservoir’s size. Specific taxa, including *Roseburia*, have also been implicated in the efficacy of vedolizumab (anti-α4β7 antibody) therapy during IBD [47]. In the light of these previous findings, we sought to determine whether differences in SCFA generation may contribute to differential responses to anti-α4β7 antibody during SIV infection. Consistent with the current paradigm, acute SIV infection was associated with a decrease in acetate and butyrate (as well as a non-significant decrease in propionate). However, in contrast to our current understanding, all three SCFAs increased following cART initiation and viral control. Importantly, this increase includes butyrate, which is frequently implicated in the immunological response to HIV/SIV infections. Notably, butyrate was also the SCFA with the most significant proportional loss, which was subsequently regained during cART. Thus, the former finding of lost butyrate confirms many findings of HIV pathogenesis in the SIV-infected NHP model.

To better understand how microbiome composition influences SCFA generation, we next performed linear regression analysis to determine which taxa were most closely associated with levels of individual SCFAs at each time point. Because the microbiome is so diverse, we did not expect to see many associations, and many discovered associations were unexpected. Significantly, *Faecalibacterium* was found to be associated with levels of SCFAs, including butyrate, the SCFA that is commonly reported in the literature. However, *Prevotella* was also frequently associated with SCFAs, including butyrate. Also unexpected was the association of *Roseburia* with acetate and propionate, but not butyrate. As previously suggested, these findings can be attributed to the complex structure of the enteric microbiome. Synthetic microbiome modelling has indicated that the community-level influence of hydrogen sulfide, environmental pH, and resource competition significantly contribute to butyrate production *in vivo* [48]. Further, dietary fibre consumption and total microbial biomass contribute more to SCFA generation than the relative abundance of specific SCFA-producing taxa because substrate availability is a limiting factor in SCFA generation and is reasoned to contribute to the significant variability in total bacteria in the intestines.

Having characterized faecal SCFA and microbiome composition, we next examined their influence on markers of SIV pathogenesis. Although there was no significant difference in VLs between the two groups, linear regression analysis was performed to test whether SCFAs influenced VLs, as suggested by *in vitro* data [49]. Following VL rebound, the SCFAs measured during cART were predictive of subsequent measures of VL at rebound setpoints, with significance in the values of samples obtained on day 98. This interrelationship included the faecal acetate concentration, which was negatively associated with VLs on days 128, 140, and 161 (the last day measured for all RMs), as well as duodenal tissue VL at necropsy. In comparison, day 98 faecal propionate only predicted day 128 VL significantly (the trend still existed for days 140, 161, and the duodenum with *p* < 0.1). In contrast, butyrate was never significantly associated with subsequent VLs, a finding contrary to our hypothesis. We also performed linear regression on acetate, propionate, and butyrate added together to determine whether total SCFAs (of those we measured) were predictive of VL. This analysis yielded the same significant associations as acetate, a finding that is perhaps unsurprising given the relatively high acetate concentration compared to the other SCFAs. Intriguingly, though, acetate, unlike other SCFAs, does not induce HIV transactivation when cells are treated with a single SCFA [49]. However, it increases transactivation when administered with other SCFAs, such as butyrate and propionate. Additionally, acetate has been shown to induce cellular anti-viral responses against respiratory syncytial virus (RSV) [50,51]. These findings contrast those on butyrate levels, which have been heavily implicated in increased viral transcription and replication by means of HDAC inhibition, a result consistent with the lack of statistical association between viral control and faecal butyrate concentrations in our study [49]. In the context of cART, HDAC inhibition may facilitate improved clearance of viral reservoirs by inducing viral transcription, thereby promoting adaptive anti-viral immune responses (consistent with the so-called “shock and kill” cure strategies) [52]. However, in the absence of cART, as in the latter part of our study, butyrate and propionate may increase viral clearance and replication concurrently, thus generating noise in our data and inhibiting clear trends in statistically significant negative associations. Notably, despite effective suppression, two RMs in our study had detectable viremia prior to ATI as measured by PCR assay detection limits (<100 copies/ml). Although the measured SCFAs at this time point were within a similar range as the other RMs, we could not determine whether they played a role in viral reactivation. Future studies should interrogate the role SCFAs play in incomplete viral suppression, as well as long-term viral suppression or without viral suppression. In light of these findings, though, acetate may be a superior immune-modulating SCFA compared to butyrate and propionate, as acetate can increase anti-viral pathways without inhibiting HDAC or inducing pro-tolerogenic phenotypes in CD103+ DCs. These data strongly implicate SCFAs in viral control in the SIV infection model.

Recently, intestinal damage in NHP models of partial body radiation-induced mucosal injury significantly decreased gut atRA concentrations, which are closely associated with the loss of circulating plasma atRA concentrations [53]. This atRA loss was further related to histological damage similar to HIV/SIV infection, including epithelial barrier dysfunction, inflammation, and villus blunting [53]. The finding that plasma atRA is rapidly depleted during SIV infection, a pattern confirmed in our study, is thus consistent with current models implicating gut damage as a primary mechanism of HIV/SIV pathogenesis [1,2,40]. More recently, it has been verified that HIV acquisition in humans is associated with decreases in plasma and duodenal atRA levels [22]. However, unlike SIV-infected macaques and PLWH, SIV-infected natural hosts such as SMs have elevated levels of atRA [20]. Previously, it was shown that administration of anti-α4β7 mAb led to an increase in plasma atRA in SIV-infected macaques [40]. Although the precise mechanism remains unknown, this increase was associated with viral control, suggesting that undetectable VL may be required to return to homeostasis. However, based on the data presented herein, we suggest that anti-α4β7 can facilitate this rebound even without viral control, as we observed higher atRA even in RMs with incomplete viral suppression during cART. This finding is consistent with natural, non-progressing SIV hosts, which can initiate gut healing despite chronic viremia [21]. In addition to its role in gut homeostasis, atRA, as well as synthetic analogues, have been proposed as latency-reversing agents promoting apoptosis in infected CD4+ T cells, thereby reducing the size of the viral reservoir [22,54–56]. However, this process may necessitate the presence of CD8+ T cells, which were experimentally depleted before infection in the present study [57]. Additionally, loss of atRA homeostasis has been implicated in cytotoxic CD8+ T cell dysfunction in models of colorectal cancer, further suggesting a role for CD8+ T cells in the efficacy of anti-α4β7 therapy in facilitating viral control [58].

To better understand the role of the gut in modulating systemic retinoid metabolism, we determined the expression levels of several enzymes related to atRA synthesis and genes that are upregulated in response to atRA. Despite the lack of significant differences in the tissue samples from the duodenum or ascending colon, the data obtained suggest that current models of the function of atRA in gut inflammation have thus far been oversimplistic. Namely, we did not observe increases in CD103+ DCs or ALDH1A2 expression, which are canonical markers of pro-tolerogenic atRA signalling in the gut, in either the duodenum or ascending colon tissues [22,46]. Additionally, it seems unlikely that the gut epithelium is driving changes in levels of atRA since ALDH1A1 was not significantly different between the two groups [46]. Despite this finding, atRA levels were still increased by anti-α4β7 mAb administration in both the small and large intestines and the periphery, indicating that the mechanism associated with increased levels may still depend on a return to gut homeostasis. One possibility is that mature lamina propria macrophages could directly mediate increased atRA synthesis, as suggested by the correlation between plasma and gut atRA with co-expression of CD206 with CD163 on cells within the duodenum [25]. However, atRA is required for the expression of ILC1 and ILC3 gut-homing receptors; thus, migration to the intestine provides a second possible mechanism [59]. Previous studies have indicated that anti-α4β7 mAb may facilitate the return of ILCs to the gut, and ILC3s are specifically required to produce GM-CSF in the gut mucosa [40,60]. Increased numbers of ILC3s could thus facilitate increased monocyte homing, differentiation, and maturation of lamina propria macrophages [61]. Therefore, the association between atRA synthesis and mature macrophage phenotypes may be indirectly mediated by ILCs and requires further investigation. An additional possibility is that anti-α4β7 further inhibits the catabolism of atRA, such as the reaction facilitated by CYP26A1, a gene not examined in this study [62]. Our data did not support this hypothesis, though, as we observed a decrease in RE following the administration of anti-α4β7. Therefore, complete elucidation of the entire retinoid metabolism network relevant to SIV/HIV pathogenesis will require further analysis of additional pathways in tissue, cellular, and enzymatic components.

In contrast to the association between SCFAs and the magnitude of VL rebounds after cART interruption, the correlations between ALDH1A2 were more consistent with previous literature, which suggested that butyrate can induce ALDH1A2 expression in CD103+ dendritic cells [44,45]. We found that butyrate in samples collected at day 98, but not other SCFAs, was associated with subsequent ALDH1A2 expression in the large intestine at necropsy. Butyrate was also positively associated with ALDH1A2 in samples on day 161. The close association between SCFAs and retinoid metabolism in the ascending colon, but not in the duodenum, is consistent with the fermentation of dietary fibre, which occurs more distally in the gastrointestinal tract and primarily in the large intestine. Thus, our findings provide a rationale for the local effect of anti-α4β7 antibody in significantly modulating small intestine gut macrophage maturity; metabolites, microbial antigens, and pattern recognition ligands likely influence myeloid cells in the large intestine more considerably due to microbial enrichment at this anatomical location [25].

Our studies have highlighted certain limitations, including fewer animals in study groups, the need for longer-term viral suppression, and the absence of animals subjected to acute CD8+ depletion. To address these limitations, we recommend for additional investigations. Utilizing techniques such as single-cell RNA sequencing (scRNA-seq) of tissues and peripheral blood can enhance our understanding of cell subset distribution and RA signaling per cell subset. Unbiased metabolomics is crucial for quantifying other metabolites that may undergo alterations. Furthermore, incorporating plasma pathogen sequencing and assessing microbial translocation markers like sCD14 and LPS can provide valuable insights into the microbiome’s role in crosstalk with the immune system. These comprehensive approaches are necessary to understand how the microbiome influences gut homeostasis following anti-α4β7 antibody and will inform future studies to shed further light on the complex mechanisms in these scenarios.

## Conclusions

The microbiome has been implicated in HIV/SIV pathogenesis and other diseases associated with gut dysregulation, such as IBDs. However, 16S sequencing may not be sufficient for ongoing efforts to characterize its role in disease modulation, as our findings implicate SCFAs in relation to total microbiome composition and not necessarily a particular taxon as a primary determinant of disease outcome measures. Further, when attempting to replicate studies across institutions, new efforts may be needed to re-evaluate experimental inputs, such as dietary composition, especially dietary fibers. The data presented herein reinforce previous findings regarding the role of the microbiome and its metabolites, namely SCFAs, thereby offering new targets for experimental intervention, as well as confounders that should be further characterized, especially when determining the efficacy of strategies to modulate mucosal immune responses such as anti-α4β7 monoclonal antibody administration.

## Supplementary Material

Supplemental Material

## Data Availability

16S rRNA sequencing FASTQ files are available in the previously uploaded BioProject in the NCBI Sequence Read Archive under the accession number PRJNA870961. The software and reagents used in this study are included in the Materials and Methods section.
