# Supplementary material for: Dual role for microbial short-chain fatty acids in modifying SIV disease trajectory following anti-α4β7 antibody administration
Source: Ann Med. 2024 Feb 14;56(1):2315224. doi: 10.1080/07853890.2024.2315224 (PMC10868432; doi:10.1080/07853890.2024.2315224)
Supplement: Supplemental Material [file IANN_A_2315224_SM1802.docx]

**Supplemental Materials**

**Dual Role for microbial short-chain fatty acids in modifying SIV disease trajectory following anti-α4β7 administration**

Samuel D. Johnson^1,2^, Nageswara Pilli^3^, Jianshi Yu^3^, Lindsey A. Knight^2^, Maureen A. Kane^3^, and Siddappa N. Byrareddy^2,4,5^

1. Department of Pathology and Microbiology, University of Nebraska Medical Center, Omaha, NE, USA
2. Department of Pharmacology and Experimental Neuroscience, University of Nebraska Medical Center, Omaha, NE, USA
3. Department of Pharmaceutical Sciences, University of Maryland School of Pharmacy, Baltimore, MD, USA
4. Department of Genetics, Cell Biology and Anatomy, University of Nebraska Medical Center, Omaha, NE, USA
5. Department of Biochemistry and Molecular Biology, University of Nebraska Medical Center, Omaha, NE, USA

**Supplemental Figure 1:** 16S rRNA sequencing for longitudinal fecal microbiomes comparing control and anti-α4β7-treated RMs.


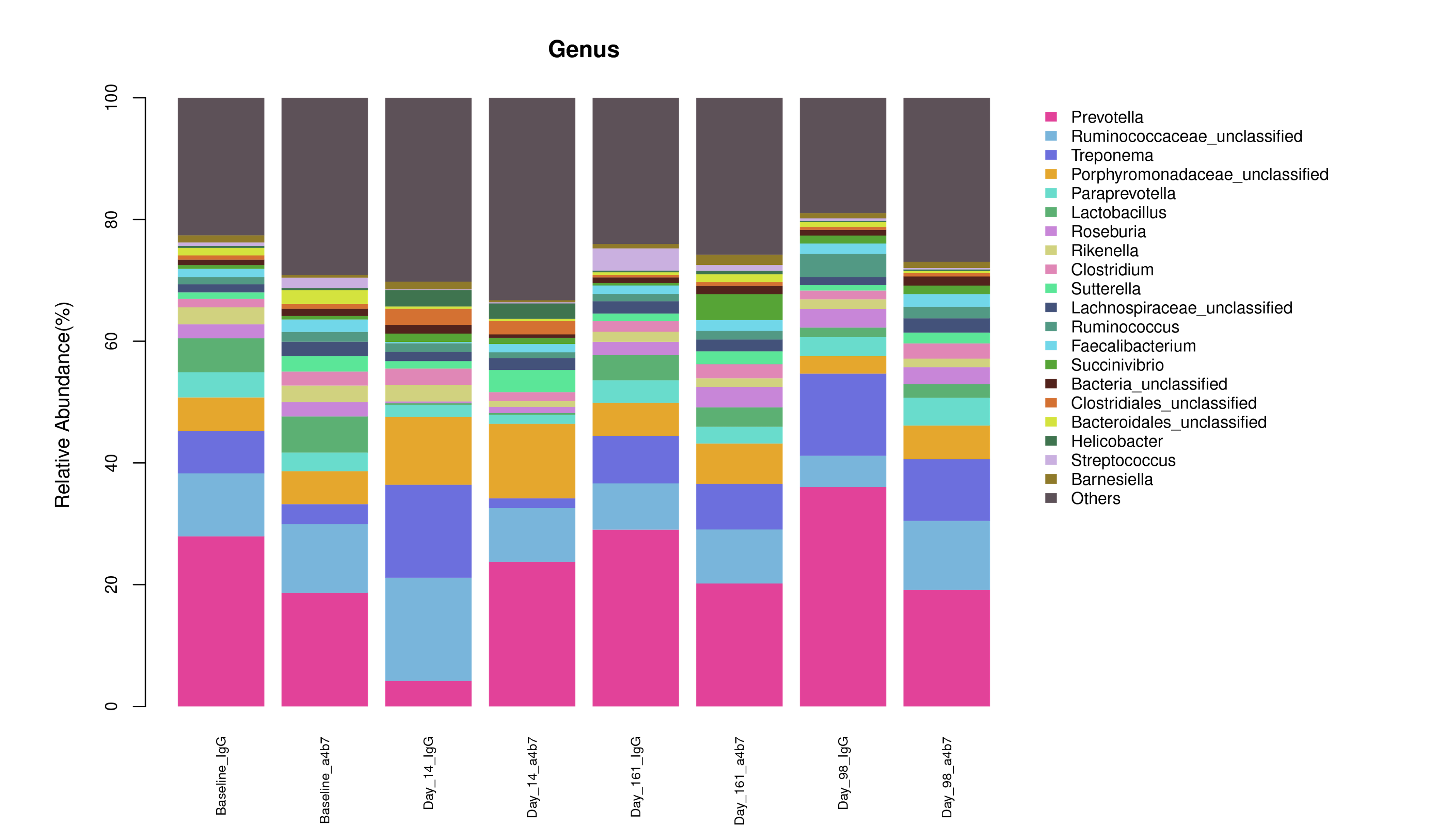


**Supplemental Figure 2**: Anti-α4β7 does not facilitate viral control in CD8-depleted rhesus macaques. Longitudinal plasma viral loads were determined with qRT-PCR and reported in copies/mL.


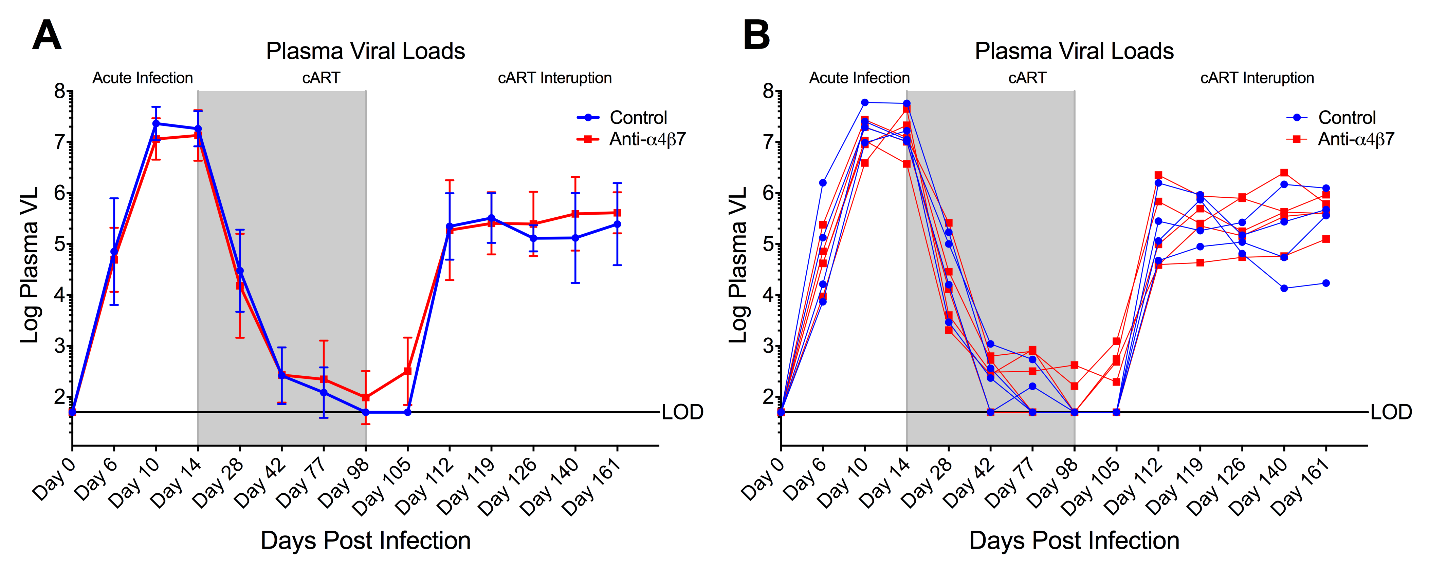


**Supplemental Figure 3:** Plasma and tissue atRA are markers of macrophage maturity in the duodenum. Previously reported co-localization of CD206 with CD163 (Mander’s Coefficient) was positively associated with plasma atRA at days 98 and 161 and tissue atRA at necropsy.


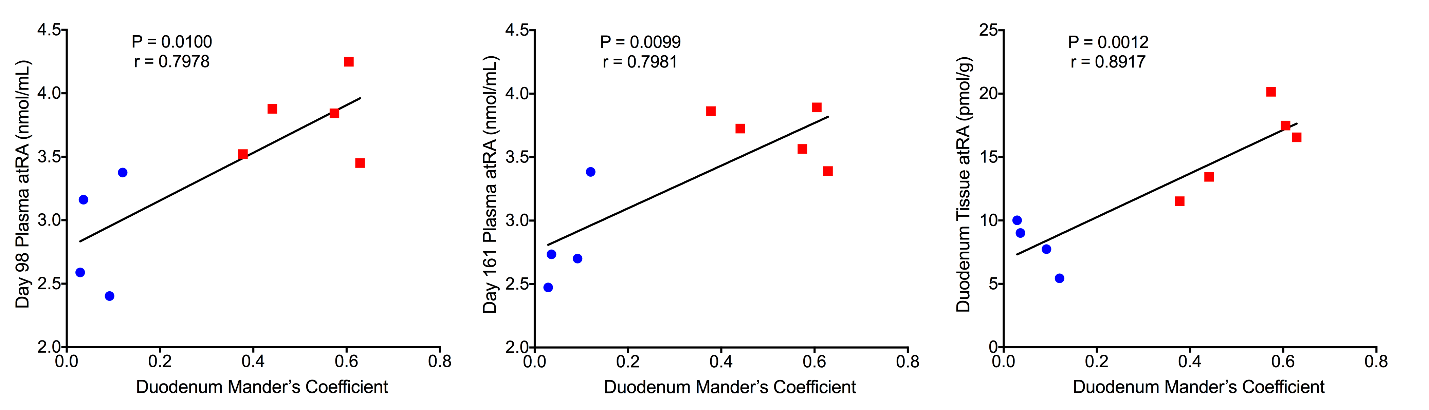


**Supplemental Figure 4**: Anti-α4β7 administration is not associated with significant changes in retinoid metabolism genes. Relative expression of atRA synthesis genes RDH10, ALDH1A1, ALDH1A2, and ALDH1A3 and atRA response genes RAR-a, RAR-b, RAR-c, CRABP2, and TGM2 were determined at necropsy for the (A) duodenum and (B) ascending colon.


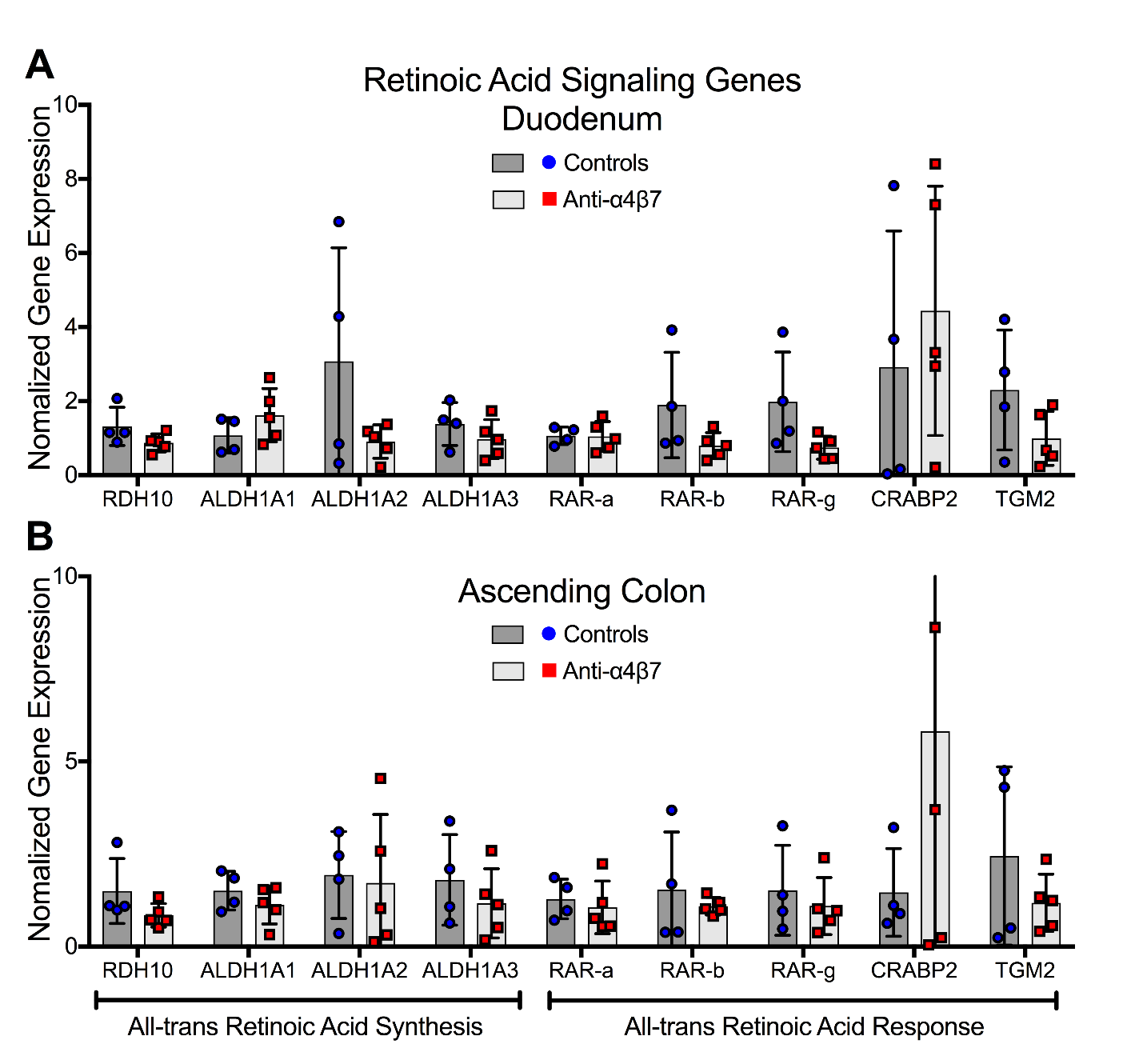


**Supplemental Table 1**: Primer sequences

**Supplemental Table 2**: Pearson coefficients and P-values derived from linear regression analysis for associations between fecal SCFA and the relative abundance of fecal microbiome genera. Each SCFA and genus was compared only with the data collected from the same timepoint.

**Supplemental Table 3**: Pearson coefficients and P-values derived from linear regression analysis for associations between plasma retinoids and gut tissue atRA synthesis and response genes.
